# Alpinumisoflavone ameliorates experimental acute reflux esophagitis in rats via regulation of inflammatory pathway

**DOI:** 10.1590/acb405625

**Published:** 2025-10-27

**Authors:** Zhaorigetu, Zi Ge, Wei Zhang

**Affiliations:** 1 The Affiliated Hospital of Inner Mongolia University for Nationalities – Department of Gastroenterology – Tongliao Inner Mongolia – China.; 2 Baoshan branch of Huashan Hospital Affiliated to Fudan University – Department of Intensive Care Unit – Shanghai – China.; 3 Shaanxi Province Hospital of Traditional Chinese Medicine – Department of The First Spleen-stomach Disease – Shaanxi – China.

**Keywords:** Esophagitis, Peptic, Inflammation, Antioxidants, Disease Models, Animal, NF-kappa B

## Abstract

**Purpose:**

To scrutinize the protective effect of alpinumisoflavone against the acute reflux esophagitis (RE) in the rats and to explore the underlying mechanism.

**Methods:**

RAW 264.7 cells were used for *in-vitro* study, and MTT assay was used to access the cell viability. The cells were treated with lipopolysaccharide (LPS) and estimation the inflammatory cytokines and parameters. A surgical procedure was performed for the induction of RE followed by the oral administration of alpinumisoflavone (5, 10 and 15 mg/kg). The esophagitis lesion score, gross esophageal score, damage ratio, pH and gastric volume, NO level, alcian blue, H_2_O_2_, free iron, calcium, antioxidant, inflammatory cytokines and inflammatory parameters were estimated.

**Results:**

Alpinumisoflavone treatment significantly (*p* < 0.001) suppressed cell viability and NO levels, along with a reduction in inflammatory cytokines like tumor necrosis factor (TNF)-α, interleukin (IL)-1β, IL-6 and inflammatory parameters such as cyclooxygenase-2 (COX-2), prostaglandin E2_2_ (PGE_2_), and inducible nitric oxide synthase (iNOS) against the LPS treatment. Alpinumisoflavone treated group rats suppressed the esophagitis lesion score, gross esophageal score damage ratio, and gastric volume and improved the pH level. Alpinumisoflavone treatment significantly (*p* < 0.001) suppressed the level of NO level, alcian blue, H_2_O_2_, free iron and calcium. Alpinumisoflavone significantly (*p* < 0.001) altered the level of antioxidant parameters such as malondialdehyde, superoxide dismutase, glutathione, glutathione peroxidase, catalase; inflammatory cytokines viz., TNF-α, IL-6, IL-1β, IL-10, IL-18; and inflammatory parameters including COX-2, NF-κB, and PGE2.

**Conclusion:**

Alpinumisoflavone ameliorates the acute reflux esophagitis via suppression of inflammatory parameters.

## Introduction

Reflux esophagitis (RE) is a prevalent gastrointestinal condition that affects a significant portion of the global population, with an estimated 40% of individuals experiencing symptoms. This disorder occurs when the protective mechanisms that prevent acid reflux fail, leading to excessive exposure of the esophagus to gastric juice^1^. The significant factor in the development of RE is gastric acid, which plays a crucial role in its pathogenesis. In patients with RE, abnormal relaxation of the lower esophageal sphincter (LES) allows acidic contents to flow back into the esophagus. The chronic RE can be severe and potentially lead to long-term complications^2^,^3^. These include chronic esophagitis, characterized by persistent inflammation of the esophageal lining, as well as the formation of esophageal strictures, which are narrowed sections of the esophagus that can cause difficulty swallowing. Additionally, RE can progress to Barrett’s esophagus, a condition in which the normal squamous epithelium of the esophagus is replaced by intestinal or columnar metaplasia. This transformation is considered a precursor to esophageal adenocarcinoma, highlighting the importance of proper management and treatment of RE^2^–^4^.

It is worth noting that while gastric acid is the primary contributor to esophageal damage, other components of gastric contents may also play a role in causing chemical injury to the esophageal tissue, further emphasizing the complex nature of this disorder. The reflux of gastric acid and stomach contents into the esophagus is a complex pathophysiological process that can lead to significant tissue damage and inflammation^5^. When this reflux occurs, the esophageal epithelium is exposed to harsh acidic conditions and digestive enzymes, resulting in cellular injury and death. This damage triggers a cascade of inflammatory responses, characterized by the infiltration of various inflammatory cells such as neutrophils, macrophages and lymphocytes. These cells release pro-inflammatory cytokines and other mediators, further exacerbating the tissue damage and perpetuating the inflammatory state^6^–^8^.

The chronic nature of this process can lead to persistent symptoms and long-term complications associated with gastroesophageal reflux disease (GERD). While current therapeutic approaches for GERD, including histamine-2 receptor antagonists, proton pump inhibitors and antacids, aim to reduce acid production and neutralize stomach contents, they have limitations in addressing the full spectrum of the disease^9^,^10^. These treatments primarily focus on symptom relief and acid suppression, but may not adequately address the underlying tissue damage or prevent recurrence. Consequently, patients may experience incomplete mucosal healing, leading to a high rate of symptom recurrence upon treatment discontinuation. Furthermore, in some cases, chronic inflammation and repeated cycles of injury and healing can lead to complications such as esophageal strictures, which further compromise esophageal function and quality of life. These limitations underscore the necessity for more comprehensive treatment strategies that not only manage symptoms but also promote tissue repair and prevent long-term complications associated with GERD^4^,^8^,^11^.

Nowadays, proton pomp inhibitors such as omeprazole or H_2_ receptor antagonists are considered as conventional anti-reflux treatments to reduce gastric acidity. They exhibit various side effects such as impotence, gynaecomastia, hypergastrinemia and hemopoietic hematopoietic changes, and ulcer relapse after long-term treatment. In recent years, developing of drugs with herbal origin is considered for decreasing the side effects of chemical drugs^6^,^8^,^12^.

 Alpinumisoflavone is a natural compound found in certain plants, especially those belonging to the Alpinia family^13^,^14^. One well-known plant in this family is *Alpinia galanga*, commonly called greater galangal. The researchers are interested in studying alpinumisoflavone because it may have beneficial effects on health. This compound belongs to a group of plant-based chemicals called isoflavonoids, which are known for their potential medicinal properties^13^–^16^. Researchers are exploring alpinumisoflavone to understand if it could be useful in developing treatments or medicines^13^,^14^,^17^. The fact that it occurs naturally in plants makes it an intriguing subject for scientific investigation.

In this experimental study, we aimed to explore the protective effect of alpinumisoflavone against the acute RE and the underlying mechanism.

## Methods

### Cell culture

For the cell culture, the RAW 264.7 cells were used. The cells were brought from the American Type Culture collection (ATCC, Rockville, MD, United States of America) and were supplemented in Dulbecco’s modified eagle medium (DMEM) with fetal bovine serum (FBS) (10%) and P/S (1%) in CO_2_ incubator chamber (SANTO, Sakata, Japan). The cells were cultured for one week and replaced the medium on alternate days. The cultured cells pre-treated with the different concentration of alpinumisoflavone for 1 h and after that incubated with lipopolysaccharide (LPS) (1 μg/mL) for 24 h.

### Cell viability

The 96-well plate were used for the cell viability test. The 6-well plate (5 × 10^5^ cells/well) and 96-well plates (1 × 10^6^ cells/well) were treated with the different concentration of alphiumisoflavone, followed by co-treated with 1 μg/mL and incubated for 24 h. The cell viability was estimated using the cytotoxicity assay kits via following the manufacture instruction.

### Nitric oxide production, inflammatory cytokines and inflammatory parameters

The culture cells were centrifuged at 2,500 rpm for 10 min and collected the supernatant of cell culture (50 μL) and mixed in the NED (0.1%), and sulfanilamide (1%) and re-incubated for 10 min at 24°C. The enzyme-linked immunosorbent assay (ELISA) reader was used for the estimation of absorbance at 540 nm (Mutiscan spectrum, United States of America). The nitric oxide (NO) level, inflammatory cytokines, and inflammatory mediators level were estimated using the manufacture instruction.

### Experimental rodent

Male Sprague Dawley rats (8–10 weeks old, body weight: 180 ± 20 g) were used in this experimental study. The rats were received from the institutional animal house and kept in the single polyethylene cage under the standard laboratory condition like 20 ± 5 °C, 60–70% relative humidity, and 12/12-h dark/light cycle. During the experimental study, the rats received the standard pellet diet and water *ad libitum*. The research was carried out in the Department of Gastroenterology, Affiliated Hospital of Inner Mongolia University, China, in the month of March 2024.

### Experimental protocol

The rats were kept in the laboratory for seven days for acclimatization and after that divided into following groups:

- Group A: normal control;
- Group B: RE;
- Group C: RE + alpinumisoflavone (5 mg/kg);
- Group D: RE + alpinumisoflavone (10 mg/kg);
- Group E: RE + alpinumisoflavone (15 mg/kg);
- Group F: RE + ranitidine (40 mg/kg),.

The dose of the tested drug alpinumisoflavone^18^ and ranitidine (40 mg/kg)^19^ were selected on the basis of previous reported protocol. For the induction of RE, surgical procedures carried out under the general anesthesia–intraperitoneal administration of ketamine and xylazine (55 and 7 mg/kg). The pyloric stenosis model was used following the previous reported method with minor modification^20^. Briefly, the duodenum near the pylorus was wrapped with a piece of Nelaton catheter (size = 4 mm), and the catheter was surrounded and tied with the sild thread (3-0) to prevent dislodgement. Using silk thread, the area connecting the glandular section to the forestomach was tied off. Rats in the sham control group underwent only a midline laparotomy without any additional surgical procedures.

After successfully inducing the RE in the rats, the rats were free from food for 48 h, but allowed to free access to drinking water. After the 48 h, the drugs (test and standard) or vehicle was given to the rats for the next 12 days. After the experimental period, the rats were fasted overnight and scarified. The whole esophagus was successfully isolated and scrutinized for the gross mucosal damage. The esophageal tissue was quickly removed and stored at -80 °C for further biochemical estimation.

### pH

After sacrificing the rats, the stomach of the all-group rats was immediately removed and rained with the NaCl (0.9%) using the micropipette (1,000 µL). The pH meter was used for the estimation of pH of the collected gastric juice.

### Gastric wall mucus level

Kitagawa et al.^21^ reported method was used for the estimation of gastric wall mucus level with minor modification. Briefly, the stomach was removed and weighed and immersed in Alcian Blue (0.1% W/V) for 2 h, and excessive dye was successfully removed via two successive washing for 15 min in the sucrose solution (0.25 M). For the removal of the excessive dye, the stomach was washed with the MgCl_2_ (0.5 M) for 2 h with intermittent shaking. The blue extract was shaken vigorously with an equal volume of diethyl ether and centrifuged for 5 min at 500 g/rpm and finally estimated the optical density of the supernatant using the spectrophotometer at 580 nm. The level of gastric wall mucus was calculated as per gram of wet stomach.

### Histamine level and H^+^-K^+^-ATPase activity 

After collecting the plasma, the plasma treated with 0.2 M perchloric acid and centrifuged for 30 min at 10,000 g/rpm at 4°C. Finally, the high-performance liquid chromatography (HPLC) was used for the estimation of histamine level.

Previous reported method was used for the estimation the activity of H^+^-K^+^-ATPase with minor modification^22^.

### Calcium, H_2_O_2_ and iron level 

Previous reported method was used for the estimation the level of calcium, H_2_O_2_ and iron level^6^.

### Myeloperoxidase and NO

The commercially available kits were used for the estimation of NO (Cayman Chemical, United States of America) and MPO (BioVision, CA, United States of America) following the manufacture instruction.

### Antioxidant parameters

The antioxidant parameters such as catalase (CAT), malondialdehyde (MDA), superoxide dismutase (SOD), glutathione peroxidase (GPx), glutathione (GSH), and sulfhydryl level were estimated using the previous reported method with minor modification^23^,^24^.

### Inflammatory cytokines and inflammatory parameters

The inflammatory cytokines such as tumor necrosis factor (TNF)-α, interleukin (IL)-1β, IL-6, IL-10, IL-17; and inflammatory parameters like cyclooxygenase-2 (COX-2), nuclear factor-kappa B (NF-κB), and prostaglandin E_2_ (PGE_2_) were estimated suing the enzyme linked immunosorbent assay (ELISA) kit via following the manufacturer instruction.

### Statistical analysis

GraphPad Prism software (St. Luis, United States of America) was used for the statistical analysis. Turkey’s t multiple comparison test was used for the statistical analysis, and *p* < 0.05 was consider as significant.

## Results

### Cell viability and NO production

**Figure 1:**
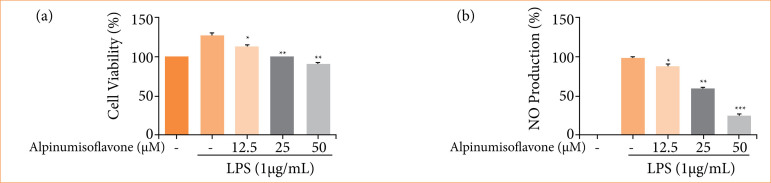
Effect of alpinumisoflavone on the cell viability and NO level on the RAW 264.7 cells. **(a)** Cell viability and **(b)** NO production. All the data are presented mean ± standard deviation.

For the estimation the effect of alpinumisoflavone on the cell growth, the RAW cells were treated with the different concentration of alpinumisoflavone for 24 h (Fig. 1a). The alpinumisoflavone treatment remarkably did not show any effect on the growth of the normal cells. After the administration of LPS, the production of NO started. It was boosted in the without treatment groups, and alpinumisoflavone remarkably suppressed the NO production (Fig. 1b) at a dose-dependent manner.

LPS: lipopolysaccharide; *p < 0.05 and **p < 0.01 compared with the lipopolysaccharide treated cells. Source: Elaborated by the authors.

### Inflammatory parameters

**Figure 2:**
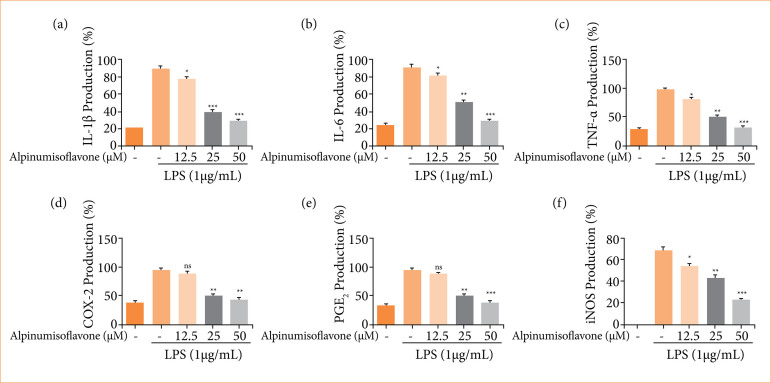
Effect of alpinumisoflavone on the inflammatory cytokines and inflammatory parameters on the RAW 264.7 cells. **(a)** IL-1β, **(b)** IL-6, **(c)** TNF-α, **(d)** COX-2, **(e)** PGE2 and **(f)** iNOS. All the data are presented mean ± standard deviation.

LPS: lipopolysaccharide; IL: interleukin; TNF: tumor necrosis factor; COX-2: cyclooxygenase-2; PGE_2_: prostaglandin E_2_; iNOS: inducible nitric oxide synthase; *p < 0.05 and **p < 0.01 compared with the lipopolysaccharide treated cells. Source: Elaborated by the authors.

The cells treated with the LPS exhibited the boosted level of inflammatory cytokines such as TNF-α (Fig. 2a), IL-1β (Fig. 2b), IL-6 (Fig. 2c); inflammatory parameters viz., COX-2 (Fig. 2d), PGE2 (Fig. 2e), inducible nitric oxide synthase (iNOS) (Fig. 2f), and dose-dependently treatment of alpinumisoflavone treatment remarkably suppressed the level of inflammatory cytokines and inflammatory parameters.

### Esophagitis lesion score, gross esophageal score damage ratio, pH, and gastric volume

**Figure 3:**
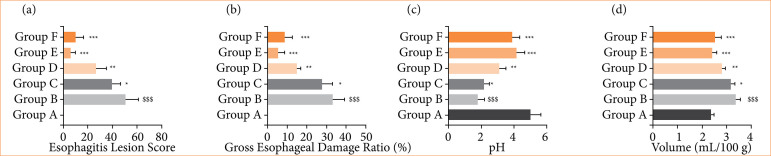
Effect of alpinumisoflavone on the esophagitis lesion score, gross esophageal damage ratio, pH, gastric volume against reflux esophagitis in rats. **(a)** Esophagitis lesion score, **(b)** gross esophageal damage ratio, **(c)** pH, and **(d)** volume. All the data are presented mean ± standard deviation.

RE group rats showed the altered level of esophagitis lesion score (Fig. 3a), gross esophageal score damage ratio (Fig. 3b), pH (Fig. 3c), and gastric volume (Fig. 3d), and alpinumisoflavone treatment significantly (p < 0.001) modulated the score of esophagitis lesion, gross esophageal score damage ratio, pH, and gastric volume.

*p < 0.05, **p < 0.01 and ***p < 0.001 compared with reflux esophagitis control group rats; ^$$$^ *p* < 0.001 compared with the normal control. Source: Elaborated by the authors.

### 3.4 NO and alcian blue

**Figure 4:**
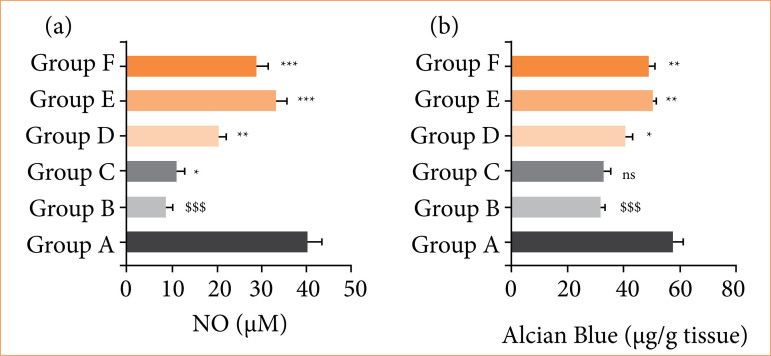
Effect of alpinumisoflavone on the level of NO and alcian against reflux esophagitis in rats. **(a)** NO and **(b)** Alcian blue. All the data are presented mean ± standard deviation.

RE group rats showed the suppressed level of NO (Fig. 4a), and alcian blue (Fig. 4b), and alpinumisoflavone treatment significantly (*p* < 0.001) improved the level of NO and alcian blue.

*p < 0.05, **p < 0.01 and ***p < 0.001 compared with reflux esophagitis control group rats; ^$$$^ *p* < 0.001 compared with the normal control. Source: Elaborated by the authors.

### 3.5 H_2_O_2_, free iron and calcium

**Figure 5:**
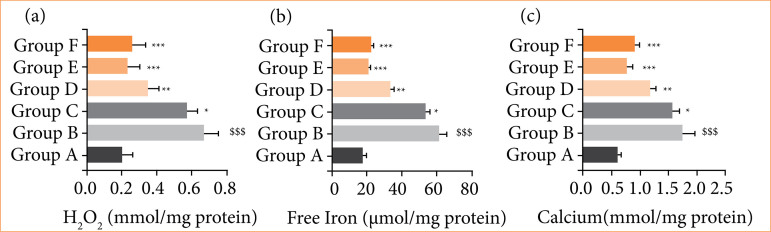
Effect of alpinumisoflavone on the level of NO and alcian against reflux esophagitis in rats. **(a)** NO and **(b)** Alcian blue. All the data are presented mean ± standard deviation.

The level of H_2_O_2_ (Fig. 5a), free iron (Fig. 5b), and calcium (Fig. 5c) reduced in the RE group ratsm and alpinumisoflavone treatment significantly (*p* < 0.001) boosted the levels.

*p < 0.05, **p < 0.01 and ***p < 0.001 compared with reflux esophagitis control group rats; ^$$$^ *p* < 0.001 compared with the normal control. Source: Elaborated by the authors.

### Antioxidant parameters

**Figure 6:**
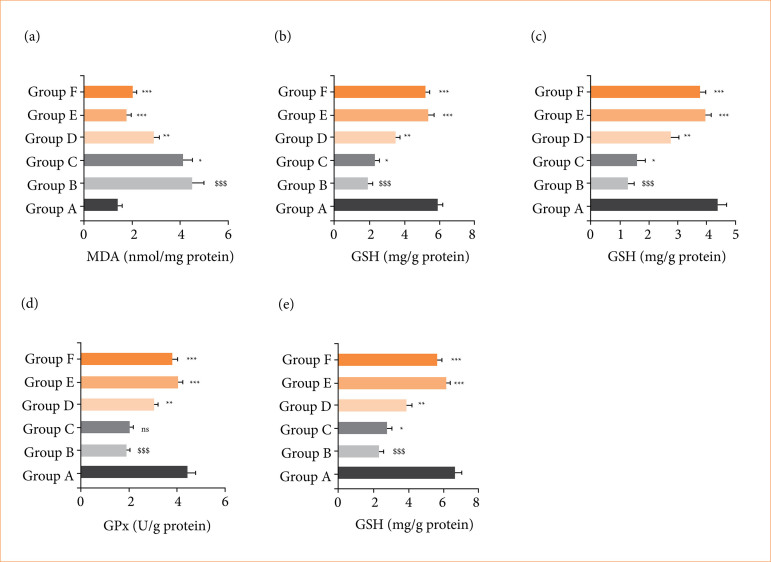
Effect of alpinumisoflavone on the level of antioxidant parameters against reflux esophagitis in rats. **(a)** malondialdehyde (MDA), **(b)** glutathione (GSH), **(c)** superoxide dismutase (SOD), **(d)** glutathione peroxidase (GPx) and **(e)** catalase (CAT). All the data are presented mean ± standard deviation.

RE group rats showed the altered level of antioxidant parameters such as MDA (Fig. 6a), GSH (Fig. 6b), SOD (Fig. 6c), GPx (Fig. 6d), and CAT (Fig. 6e), and alpinumisoflavone significantly (*p* < 0.001) restored the level of antioxidant parameters.

*p < 0.05, **p < 0.01 and ***p < 0.001 compared with reflux esophagitis control group rats; ^$$$^ *p* < 0.001 compared with the normal control. Source: Elaborated by the authors.

### Cytokines and inflammatory parameters

**Figure 7:**
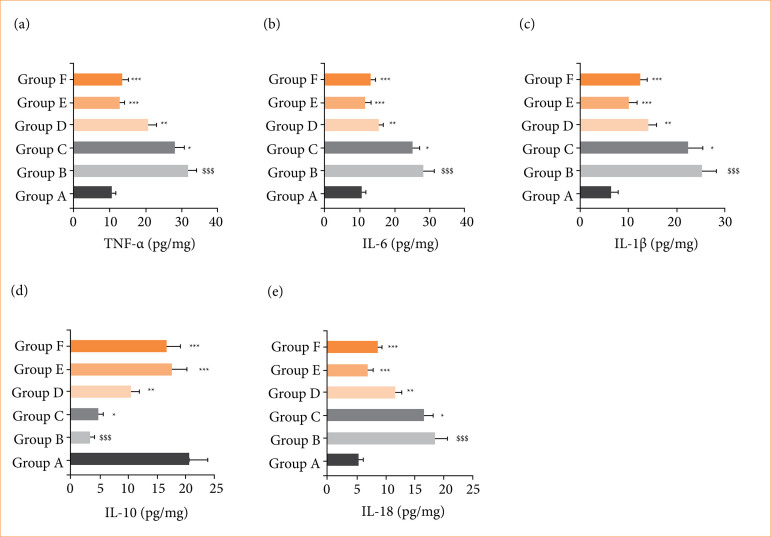
Effect of alpinumisoflavone on the level of inflammatory cytokines against RE in rats. **(a)** tumor necrosis factor (TNF)-α, **(b)** interleukin (IL)-6, **(c)** IL-1β, **(d)** IL-10 and **(e)** IL-18. All the data are presented mean ± standard deviation.

RE group rats exhibited the altered level of inflammatory cytokines including TNF-α (Fig. 7a), IL-6 (Fig. 7b), IL-1β (Fig. 7c), IL-10 (Fig. 7d), and IL-18 (Fig. 7e), and alpinumisoflavone treatment significantly (*p* < 0.001) restored the level of inflammatory cytokines.

*p < 0.05, **p < 0.01 and ***p < 0.001 compared with reflux esophagitis control group rats; ^$$$^ *p* < 0.001 compared with the normal control. Source: Elaborated by the authors.

**Figure 8:**
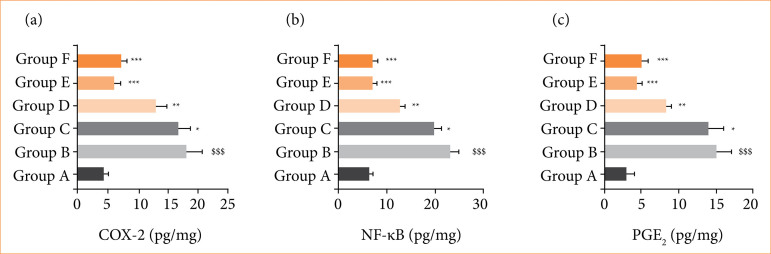
Effect of alpinumisoflavone on the level of inflammatory parameters against reflux esophagitis in rats. **(a)** cyclooxygenase-2 (COX-2), **(b)** nuclear factor-kappa B (NF-κB) and **(c)** prostaglandin E_2_ (PGE_2_). All the data are presented mean ± standard deviation.

RE group rats demonstrated the boosted level of COX-2 (Fig. 8a), NF-κB (Fig. 8b), and PGE_2_ (Fig. 8c), and alpinumisoflavone treatment significantly (*p* < 0.001) suppressed the level of inflammatory parameters.

*p < 0.05, **p < 0.01 and ***p < 0.001 compared with reflux esophagitis control group rats; ^$$$^ *p* < 0.001 compared with the normal control. Source: Elaborated by the authors.

## Discussion

GERD is a persistent disorder characterized by the recurrent backflow of gastric contents into the esophagus. This reflux is caused by the weakening or malfunction of the LES, a ring of muscle that typically serves as a barrier between the esophagus and stomach^25^,^26^. The incidence of GERD has been rising worldwide, impacting both adult and pediatric populations, with substantial effects on life quality and healthcare expenditures. Common symptoms of GERD include heartburn, regurgitation, and dysphagia, though some individuals may experience atypical manifestations such as persistent cough or chest discomfort^27^. When left untreated, GERD can result in several severe complications. Aspiration pneumonia can be developed when refluxed stomach contents enter the lungs, leading to inflammation and infection. Barrett’s esophagus, a condition in which the esophageal lining transforms to resemble intestinal tissue, is another potential long-term consequence of GERD and is linked to a higher risk of esophageal malignancy. Chronic esophagitis, or ongoing inflammation of the esophageal lining, can lead to ulceration and haemorrhage^7^,^20^. Furthermore, repeated exposure to gastric acid can result in the formation of esophageal strictures, constricting the esophagus and causing swallowing difficulties.

These complications highlight the necessity of early detection and proper treatment of GERD to prevent long-term consequences and enhance patient outcomes. RE is a multifaceted disorder with various underlying pathological processes. One proposed mechanism suggests that the esophageal mucosa’s increased sensitivity to normal reflux levels can provoke symptoms in certain individuals^3^,^28^. This enhanced responsiveness may result from changes in sensory nerve function or alterations in the mucosal barrier. Moreover, weakened mucosal defense mechanisms, such as compromised mucus production or reduced bicarbonate secretion, can render the esophageal lining more susceptible to injury from stomach contents. Abnormalities in gastric motility, including slow gastric emptying or increased transient lower esophageal sphincter relaxations, may also play a role in RE development by enhancing the frequency and duration of reflux events^20^,^29^,^30^.

Recent studies have illuminated the importance of inflammatory mediators in the pathogenesis of GERD. The referenced research indicates that GERD-related mucosal damage might be caused by inflammatory substances released from mucosal and submucosal cells in response to bile salts and gastric refluxate. This inflammatory cascade can result in tissue injury, erosion, and the typical symptoms associated with GERD.

Considering the complex nature of GERD, a more holistic treatment approach may be necessary. Rather than solely focusing on acid suppression, which has been the conventional strategy, future therapies might need to address multiple aspects of the disease. This could involve enhancing mucosal protection, mitigating inflammation, and improving gastric motility. The aim of such comprehensive treatments would be to reestablish equilibrium between aggressive factors (like acid and pepsin) and protective elements (such as mucus and bicarbonate secretion) within the esophageal environment^20^,^29^. The experimental study investigated the potential protective effects of alpinumisoflavone against acute RE in a rat model.

The highly acidic nature of stomach acid is a primary factor in causing damage to the esophageal lining. The severity of this damage is directly related to the quantity and length of time the esophagus is exposed to acid. Without proper treatment, persistent acid reflux can result in serious complications, including esophagitis, ulcers, narrowing of the esophagus, and a heightened risk of esophageal cancer^4^,^27^. This emphasizes the necessity of medical intervention and adherence to prescribed treatments for managing GERD and avoiding long-term health issues.

RE, a condition in which stomach contents flow back into the esophagus, can cause significant irritation and swelling of the esophageal mucosa. This leads to symptoms such as heartburn, pain in the chest, and difficulty swallowing^31^,^32^. The intensity of reflux esophagitis is closely linked to the acidity level and volume of the refluxed material, with higher levels of both factors leading to more severe inflammation and tissue harm. Studies have shown that during RE, there is an increase in both the amount and acidity of stomach contents, triggering inflammation in the esophagus. This connection between gastric acid properties and esophageal injury underscores the intricate pathophysiology of GERD and stresses the importance of developing targeted treatments to reduce mucosal damage and alleviate symptoms^7^,^33^.

The esophagus can become irritated and undergo morphological changes when exposed to acidic stomach fluids or regurgitated duodenal contents. This study found that esophagitis developed 2–3 cm above the esophagogastric junction, with notable lesion scores 14 days following RE surgery^34^. Alpinumisoflavone administration was shown to decrease the lesion score, suggesting its potential as a treatment for RE. Although typical reflux symptoms are linked to decreased pH and longer acid clearance times, alpinumisoflavone did not impact gastric pH levels. In comparison, omeprazole, an H^+^, K^+^-ATPase inhibitor, exhibited strong anti-secretory effects by significantly altering the pH of gastric contents.

These results are consistent with earlier research indicating that alpinumisoflavone does not interfere with gastric secretion or gastric juice pH in rats with pyloric ligatures^6^. This characteristic of alpinumisoflavone may be beneficial in addressing certain side effects associated with long-term proton pump inhibitor (PPI) use, such as hypochlorhydria or rebound hypersecretion. By maintaining gastric pH, alpinumisoflavone could offer an alternative approach to managing RE without the drawbacks of PPI-induced acid suppression. This implies that alpinumisoflavone may have a unique mechanism for alleviating esophageal damage that does not rely on gastric acid suppression, necessitating further exploration of its therapeutic potential and mode of action in treating RE.

Free radicals derived from oxygen are integral to the progression of esophageal mucosal injury caused by RE^2^,^35^. These free radicals induce mucosal inflammation, and strategies to limit their formation, such as administering superoxide dismutase, have been demonstrated to reduce RE occurrence. The heightened production of oxygen-derived free radicals is linked to increased esophageal mucosal lipid peroxidation, a sensitive indicator of free radical-induced membrane damage. This is supported by the significant elevation in lipid peroxidation levels observed in rats with RE^24^,^36^.

Moreover, glutathione, a non-enzymatic antioxidant containing thiol, is essential for facilitating the detoxification of various toxic metabolites and preserving mucosal integrity. The gastric mucosa contains high levels of glutathione, and its depletion by electrophilic compounds can result in mucosal ulceration. Oxidative stress is a critical factor in the breakdown of mucosal integrity caused by various aggressive agents^37^–^39^. The high concentration of glutathione in the gastric mucosa acts as an initial non-enzymatic defense against oxidative stress. Additionally, the enzymatic antioxidant defense system, consisting of SOD and CAT, is crucial for scavenging and regulating overall reactive oxygen species (ROS) to maintain physiological balance. Inhibiting SOD with diethyl thiocarbamate has been shown to decrease bicarbonate secretion and increase gastric lesion formation. Notably, patients with RE disease show significantly lower levels of SOD and CAT, underscoring the importance of these antioxidant enzymes in maintaining the health of esophageal and gastric mucosa^20^,^27^.

Inflammation is a key factor in the development and manifestation of gastrointestinal disorders, including GERD. Long-term acid exposure in chronic RE results in ongoing inflammatory cell infiltration and enhanced epithelial cell growth in the basal layer^8^,^9^. Research has shown increased levels of inflammatory mediators, such as TNF-α, IL-6 and IL-1β, in both animal models and human subjects with GERD. These mediators facilitate inflammatory cell recruitment, tissue injury, and changes in lower esophageal sphincter function, potentially worsening the condition. Various studies have shed light on the specific roles of these cytokines in acute reflux esophagitis^27^,^40^,^41^. TNF-α, recognized for its role in inflammation, cytotoxicity, and cachexia, has been found to enhance esophageal inflammation and tissue damage by stimulating the production of other inflammatory mediators and attracting inflammatory cells. IL-6, produced by diverse cell types, promotes esophageal epithelial cell proliferation and extracellular matrix protein synthesis, contributing to both tissue injury and repair processes. Likewise, IL-1β has been demonstrated to induce inflammation and oxidative stress in the esophagus, resulting in mucosal damage. The elevated levels of these cytokines in the esophageal tissue of rats with acute RE indicate their significant involvement in the disease’s pathogenesis^7^,^33^,^42^. These observations suggest that targeting these cytokines could be a promising therapeutic approach for treating reflux esophagitis and related gastrointestinal disorders.

The enzyme iNOS plays a crucial role in the production of nitric oxide, a molecule with multifaceted functions in the human body. While nitric oxide is essential for regulating blood flow and immune responses under normal conditions, its overproduction during inflammatory processes can have detrimental effects. The inflammation-induced upregulation of iNOS leads to excessive nitric oxide generation, which can cause tissue damage and contribute to the progression of Barrett’s esophagus and esophageal cancer. This highlights the delicate balance required in nitric oxide signaling and the potential consequences of its dysregulation in pathological conditions^7^,^43^,^44^. COX-2, on the other hand, is an enzyme involved in the biosynthesis of prostaglandin E_2_, a key mediator of inflammation and pain. The presence of COX-2 in various cell types affected by acid reflux, particularly in the epithelial cells lining the esophagus, underscores its significance in the inflammatory response to acid exposure. This enzyme’s activity contributes to the cascade of events that occur during acid reflux, potentially exacerbating tissue damage and promoting the development of esophageal disorders. The interplay between iNOS and COX-2 in the context of acid reflux and esophageal pathology demonstrates the complex network of molecular mechanisms underlying these conditions and emphasizes the importance of understanding these pathways for developing targeted therapeutic interventions^7^,^45^,^46^.

NF-κB activation in acute RE triggers a cascade of inflammatory events that contribute to the progression of the disease^47^. Upon activation, NF-κB translocates to the nucleus and binds to specific DNA sequences, promoting the transcription of various genes involved in the inflammatory response. These include genes encoding pro-inflammatory cytokines such as TNF-α, IL-1β and IL-6, as well as chemokines like IL-8 and monocyte chemoattractant protein-1 (MCP-1). Additionally, NF-κB upregulates the expression of adhesion molecules such as intercellular adhesion molecule-1 (ICAM-1) and vascular cell adhesion molecule-1 (VCAM-1), which facilitate the recruitment and infiltration of immune cells into the esophageal tissue^47^,^48^. The sustained activation of NF-κB in acute RE leads to a self-perpetuating cycle of inflammation and tissue damage. As immune cells infiltrate the esophageal mucosa, they release additional pro-inflammatory mediators and ROS, further exacerbating the inflammatory response and tissue injury. This chronic inflammation can lead to structural changes in the esophageal tissue, including epithelial cell death, mucosal erosion, and submucosal fibrosis.

Understanding the central role of NF-κB in this process has led to the exploration of targeted therapies aimed at inhibiting NF-κB activation or its downstream effectors as potential treatment strategies for acute RE^6^,^48^,^49^. These approaches may help to break the cycle of inflammation and promote tissue healing, ultimately improving patient outcomes in this condition.

## Conclusion

Alpinumisoflavone demonstrated significant anti-inflammatory and antioxidant effects in both *in-vitro* and *in-vivo* models. It effectively suppressed cell viability and nitric oxide levels, along with reducing key inflammatory cytokines (TNF-α, IL-1β, IL-6) and inflammatory markers (COX-2, PGE2, iNOS) following LPS-induced inflammation. In animal models, alpinumisoflavone significantly reduced esophagitis lesion scores, improved gastric parameters, and restored pH balance. Additionally, it modulated oxidative stress markers (MDA, SOD, GSH, GPx, CAT) and inflammatory pathways (COX-2, NF-κB), highlighting its potential therapeutic role in managing inflammatory conditions.

The limitation of this study was the underlying molecular mechanisms require further elucidation, especially regarding its interaction with the upstream signaling pathways. This study performed on the cell lines and animals, but not on the human.

## Data Availability

The data will be available on the request to the corresponding author.
